# Generation of Interstrand DNA Cross-Links under Conditions of Acid Stress

**DOI:** 10.1021/acs.chemrestox.6c00217

**Published:** 2026-06-05

**Authors:** Mithila Farzana Mumu, Marjan Heidari, Md. Selim Mahbub, Kent S. Gates

**Affiliations:** † Department of Chemistry, University of Missouri, 125 Chemistry Building, Columbia, Missouri 65211, United States; ‡ Department of Biochemistry, University of Missouri, 125 Chemistry Building, Columbia, Missouri 65211, United States

## Abstract

Bacteria encounter acid stress under a variety of circumstances. Acid stress induces DNA damage and genomic instability, most directly via acid-catalyzed depurination reactions that generate apurinic (abasic, AP) sites on the deoxyribose phosphate backbone. DNA damage responses are important in bacterial resistance to acids. A recent report provided evidence that a DNA repair glycosylase, AlkX, which is capable of initiating the repair of interstrand DNA cross-links (ICLs), contributes to acid resistance by the pulmonary pathogen *Acinetobacter baumannii* (Kunkle et al. *Proc.* Nat. Acad. Sci. USA, **2024**, 121, e2402422121). This suggested the possibility that AP-derived ICLs might contribute to the acid stress in bacteria. This idea is predicated on earlier work showing that AP sites can generate ICLs via reactions of the ring-opened AP aldehyde with the exocyclic amino groups of nucleobases on the opposing strand of duplex DNA ( PriceN. E.,  J. Am. Chem. Soc. 2014, 136, 3483 24506784 10.1021/ja410969xPMC3954461). However, it was not clear from previous work whether AP-derived ICLs could be generated under conditions of acid stress. The results reported here provide evidence for ICL formation under conditions of acid stress via a sequential process involving acid-catalyzed depurination followed by cross-linking of the resulting AP site with an adenine residue on the opposing strand of duplex DNA. This supports the possibility that AP-derived interstrand cross-links could contribute to the effects of acid stress in bacteria, and proteins involved in the repair of these lesions could be involved in resistance to acid stress.

## Graphical abstract

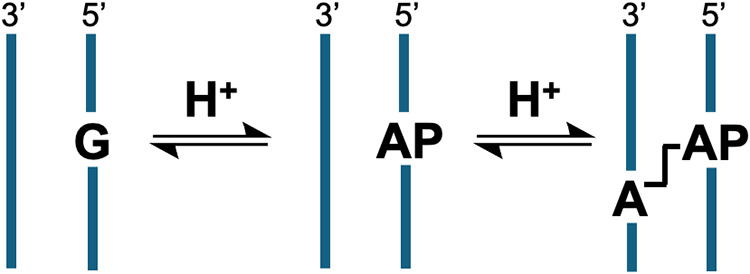

## Introduction

Acid stress is a condition experienced by bacteria in the low pH environment of the mammalian gastrointestinal tract, during interactions with host immune cells, or from the buildup of organic acids in growth media.^1,2^ The consequences of acid stress include membrane disruption, DNA damage, and alterations in protein structure and function.^1^ The mechanisms by which bacteria resist acid stress include proton pumping, acid neutralization by ammonia production, biofilm formation, and activation of DNA-damage responses.^1^ Acid stress and the mechanisms by which bacteria resist it are important in biotechnology and medicine.^1^ Interestingly, intracellular acidification also has been identified as a causative mechanism by which thymine starvation kills *E. coli*, in a well-known, but not well understood, process known as “thymineless death”.^3^

The role of DNA-damage responses in bacterial resistance to acid stress reflects the fact that acidic conditions induce DNA damage and genetic instability.^1,2,4–7^ Most directly, low pH promotes the loss of coding nucleobases from the DNA backbone via depurination and depyrimidination reactions.^8–10^ The loss of purines is much faster than the loss of pyrimidines,^8–10^ and depurination is the major pathway for DNA degradation under acidic conditions.^8–11^

Depurination reactions generate apurinic (abasic, AP) sites on the DNA backbone that can lead to genomic instability through a variety of processes including polymerase stalling, error-prone polymerase bypass, formation of DNA–protein cross-links, and formation of DNA–DNA interstrand cross-links (ICLs).^12–27^ In addition, β-elimination reactions at AP sites generate single-strand breaks^28,29^ that can lead to ICLs,^30,31^ DNA-peptide cross-links,^32–34^ DNA–protein cross-links,^24,26,35–37^ and highly toxic double-strand breaks at replication forks.^38–40^ Unrepaired AP sites in a bacterial genome are mutagenic and cytotoxic.^41,42^

A recent report characterized a DNA repair enzyme dubbed AlkX that plays a role in resistance to acid stress by the pulmonary pathogen *Acinetobacter baumannii*.^7^ Loss of AlkX resulted in increased genetic stress and reduced fitness under acidic conditions.^7^ AlkX is a DNA glycosylase that removes alkylated nucleobases from the DNA backbone and displays an unusual capacity to unhook ICLs generated by a nitrogen mustard.^7^ In this regard, AlkX joins a small group of glycosylases with the ability to initiate the repair of ICLs in duplex DNA.^43–47^

On the basis of these results, it was suggested that acid-induced ICLs might contribute to acid stress in bacteria.^7^ It is recognized that even low yields of ICLs can be exceptionally cytotoxic because these lesions prevent the strand separation required for critical DNA transactions, such as transcription and replication.^48–50^ Kunkle et al. further noted that AP sites generated by depurination reactions under conditions of acid stress could be a source of ICLs in bacterial DNA.^7^ This idea rests on earlier work^16–20^ showing that AP sites can generate ICLs via reactions of the ring-opened AP aldehyde with the exocyclic amino groups of nucleobases on the opposing strand of duplex DNA (Scheme 1). Here, we characterized the sequential process involving acid-catalyzed depurination followed by cross-linking of the resulting AP site with an opposing nucleobase in the DNA duplex under conditions of acid stress.

**Scheme 1:**
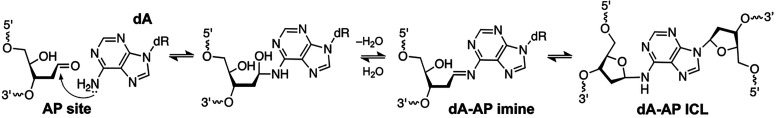
Generation of an ICL from an AP Site in Duplex DNA via the Reaction of the Ring-Opened AP Aldehyde with an Exocyclic Amino Group of a Nucleobase on the Opposing Strand of the Double Helix

## Experimental Procedures

### Materials

Oligonucleotides were purchased from Integrated DNA Technologies IDT, (Coralville, IA) and Sigma-Aldrich (St. Louis, MO). Some oligonucleotides contained a 1,1′-diethyl-2,2′-dicarbocyanine (Cy5) fluorophore on the 5′-end and were HPLC-purified before use. Uracil DNA glycosylase (UDG) was purchased from New England Biolabs (Ipswich, MA, USA). Acrylamide/bis-acrylamide (19:1, 40% solution, electrophoresis grade) was purchased from Fisher Scientific (Waltham, MA). Methoxyamine hydrochloride and buffer salts were purchased from Sigma-Aldrich (St. Louis, MO). The buffers were adjusted to the reported pH values at 24 °C. The “10×” stock solutions of phosphate-citrate “universal buffer”^51^ were prepared by dissolving disodium phosphate (1.87 g) and citric acid monohydrate (1.43 g) in DI water (25 mL). HCl was added to achieve the desired final pH and the total volume was adjusted to 40 mL by the addition of deionized water to give a final concentrations of 0.33 M phosphate and 0.17 M citric acid. The measurement of fluorescent DNA bands in polyacrylamide gels was carried out using a Fujifilm FLA 3000 (GE Healthcare) with Image Reader (v 1.12) and Image Gauge software (v 4.0).

### Characterization of Acid Depurination in DNA Duplex 1 and Single-Strand DNA Oligomer **2**

DNA duplex **1** or single-stranded DNA oligomer **2** were dissolved in pH 3.5 citrate-phosphate buffer^51^ (17 mM and 33 mM, respectively, containing 100 mM NaCl) and incubated at 37° C for 120 h (in the case of **2**) or 240 h (in the case of **1**). The DNA was ethanol precipitated,^52^ the resulting DNA pellet was briefly dried under vacuum, and the DNA redissolved in aqueous piperidine (1 M). The solution was heated at 95 °C for 30 min to induce strand cleavage at AP sites and then dried in a SpeedVac concentrator. NaOH treatment can also be used to induce strand cleavage at AP sites (Figures 3, S3, and S5). The DNA was redissolved in water (20 μL) and evaporated under vacuum three times. The DNA was redissolved in formamide loading buffer,^52^ loaded onto a 0.4 mm thick, denaturing (7 M urea), 20% polyacrylamide gel (prepared in 45 mM Tris-borate pH 8.0, containing 1 mM EDTA), and electrophoresed in Tris-borate-EDTA at 500 V for 14 h. The labeled DNA fragments in the gel were visualized and quantified by fluorescence imaging.

### ICL Formation in the AP-Containing Duplex **4**

Authentic standards for the cross-linked duplexes **5** and **6** were generated from the AP-containing duplex **4**. Specifically, the 5′-Cy5-labeled, DNA duplex **3**, containing a 2′-deoxyuridine residue at position 18 was treated with uracil DNA glycosylase (UDG, 50 units/mL) at 37 °C for 2 h to generate the AP-containing duplex **4**.^53–55^ The DNA was ethanol precipitated, and the resulting pellet briefly dried under vacuum in a SpeedVac concentrator. Generation of the AP site was confirmed by alkaline workup (200 mM NaOH, 60 °C, for 30 min) that induces strand cleavage at the AP site.^29^ In the cross-linking reactions, the DNA was redissolved in the desired buffer (either HEPES pH 7.4 or citrate-phosphate pH 3.5) and incubated for the time indicated in the Figure Legends. Incubation of the AP-containing duplex **4** in HEPES buffer (50 mM, pH 7.4, containing 100 mM NaCl) for 120 h at 37 °C generated an authentic standard for the full-length cross-linked duplex **5**.^17^ Alternatively, incubation of duplex **4** in HEPES buffer (50 mM, pH 7.4) containing NaCl (100 mM) and spermine (Sp, 2 mM)^29^ produced an authentic standard for the cross-linked duplex **6**, derived from strand cleavage at the AP site.^31^

### Depurination and ICL Formation in Duplex **1** Under Acidic Conditions

Duplex **1** was incubated in citrate-phosphate buffers (17 mM and 33 mM, respectively, containing 100 mM NaCl) at 37 °C for 120 h. The DNA was ethanol precipitated^52^ and the resulting DNA pellet was briefly dried under vacuum. The DNA was redissolved in formamide loading buffer,^52^ loaded onto a 0.4 mm thick, denaturing (7 M urea), 20% polyacrylamide gel, and electrophoresed in Tris-borate-EDTA at 500 V for 14 h. The labeled DNA fragments in the gel were visualized and quantified using Fujifilm FLA 3000 (GE Healthcare) Image reader (v 1.4) with Image Gauge (v 1.6) software. In the reactions shown in lanes 6 and 9 of Figure 3, methoxyamine was present from the beginning of the reactions. Experiments with duplex **7** in pH-3.5 citrate-phosphate or pH 7.4 HEPES buffer and duplex **1** in pH 7.4 buffer were carried out as described above.

## Results and Discussion

### Design of a DNA Duplex for the Study of Acid-Induced ICL Formation

Purines (adenine and guanine residues) are the expected locations for the formation of AP sites under acidic conditions.^8–11^ Previous work has shown that AP sites have the potential to generate ICLs at locations where an adenine residue is located one nucleotide (nt) to the 3′-side of the AP site on the opposing strand or where a guanine residue is located one nt to the 5′-side of the AP site on the opposing strand (Figures 1 and S1).^16–18,56^ In experiments near neutral pH, the equilibrium yields of the dA-AP ICL typically are higher (10–80%) than the yields of the dG-AP ICL (2–5%).^16,17,56^ We designed a mixed-sequence, 35 nt duplex **1** containing 36 purine residues (Figure 1). Of these 36 depurination sites, 25 were located in sequence environments that confer the potential for AP-derived ICL formation (indicated by arrows in Figure S1). The duplex contained an established,^17,56^ high-yield site for dA-AP cross-link formation at G18 (CXT/AAG, where X = AP).

**Figure 1:**
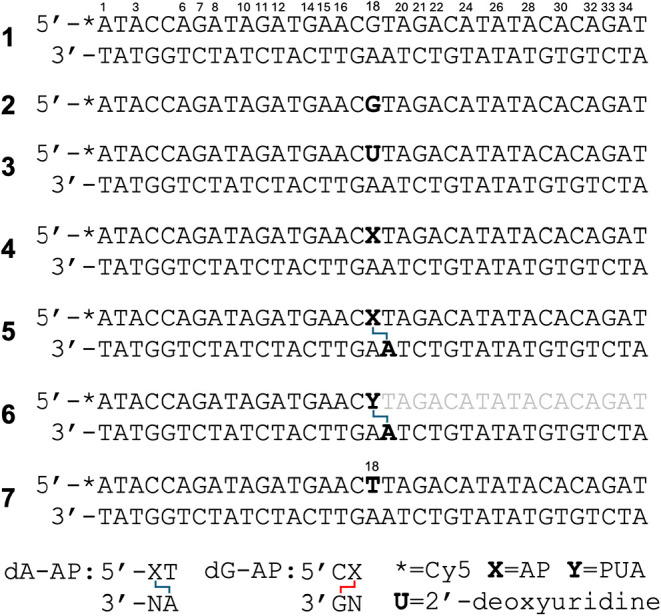
DNA sequences used in these studies. Sequence motifs that favor formation of the dA-AP and dG-AP ICLs are shown in the lower left of the Figure. Chemical structures for the X-A and Y-A cross-links are shown in Scheme 1 and Figure S1. The black/gray color discontinuity in duplex **6** is intended to highlight the presence of a strand break at “Y”.

### Acid-Catalyzed Depurination in the DNA Duplex **1** and the Single-Stranded DNA Oligomer **2**

We used gel electrophoretic analyses to characterize acid-induced generation of AP sites in duplex **1** and in the corresponding single-stranded DNA oligomer **2** at pH 3.5. The conditions may mimic the effects of an extreme acid stress in bacteria. For example, exposure of *E. coli* cells, in which inducible acid response systems are not operating, to an external pH of 2.5, such as that found in the gastrointestinal tract, leads to intracellular pH values near 3.5 and poor cell survival.^57,58^

The 35 nt DNA oligomer **2** was labeled on the 5′-end with a 1,1′-diethyl-2,2′-dicarbocyanine fluorophore (Cy5, denoted as * in Figure 1) to enable visualization of the products in the gels.^59^ Depurination events in the labeled oligonucleotide were revealed by base workup (either NaOH or piperidine) that induced strand cleavage at the AP sites via α,β-elimination reactions that generated shorter, labeled DNA fragments containing a 2-deoxyribose or phosphoryl unsaturated aldehyde (PUA) sugar remnant on the 3′-terminus.^10,29^ Subsequent γ,δ-elimination reactions result in loss of the 3′-sugar remnant to generate DNA fragments with a 3′-phosphoryl end group (3′P).^10,29^ The labeled fragments derived from cleavage of AP sites in the DNA oligomer migrate faster in the gel than the full-length, labeled 35 nt oligomer, giving bands that appear below the full-length DNA in the gel images shown here. The amount of each cleavage product in the gels, corresponding to the amount of depurination at each site, was quantitatively measured using fluorescence imaging.

We incubated the single-stranded DNA oligomer **2** in pH 3.5 buffer at 37 °C for 120 h, followed by piperidine workup to convert AP sites into strand cleavage products (Figure 2A). The total depurination yield across all sites resolved in the gel was 52%. We observed depurination at all A and G residues. Overall, we measured higher depurination yields at A residues compared to G residues, with seven of the top eight depurination hotspots seen at A residues (Figure 2B). The observed preference for depurination of A residues over G residues at low pH is consistent with a previous report.^11^

**Figure 2:**
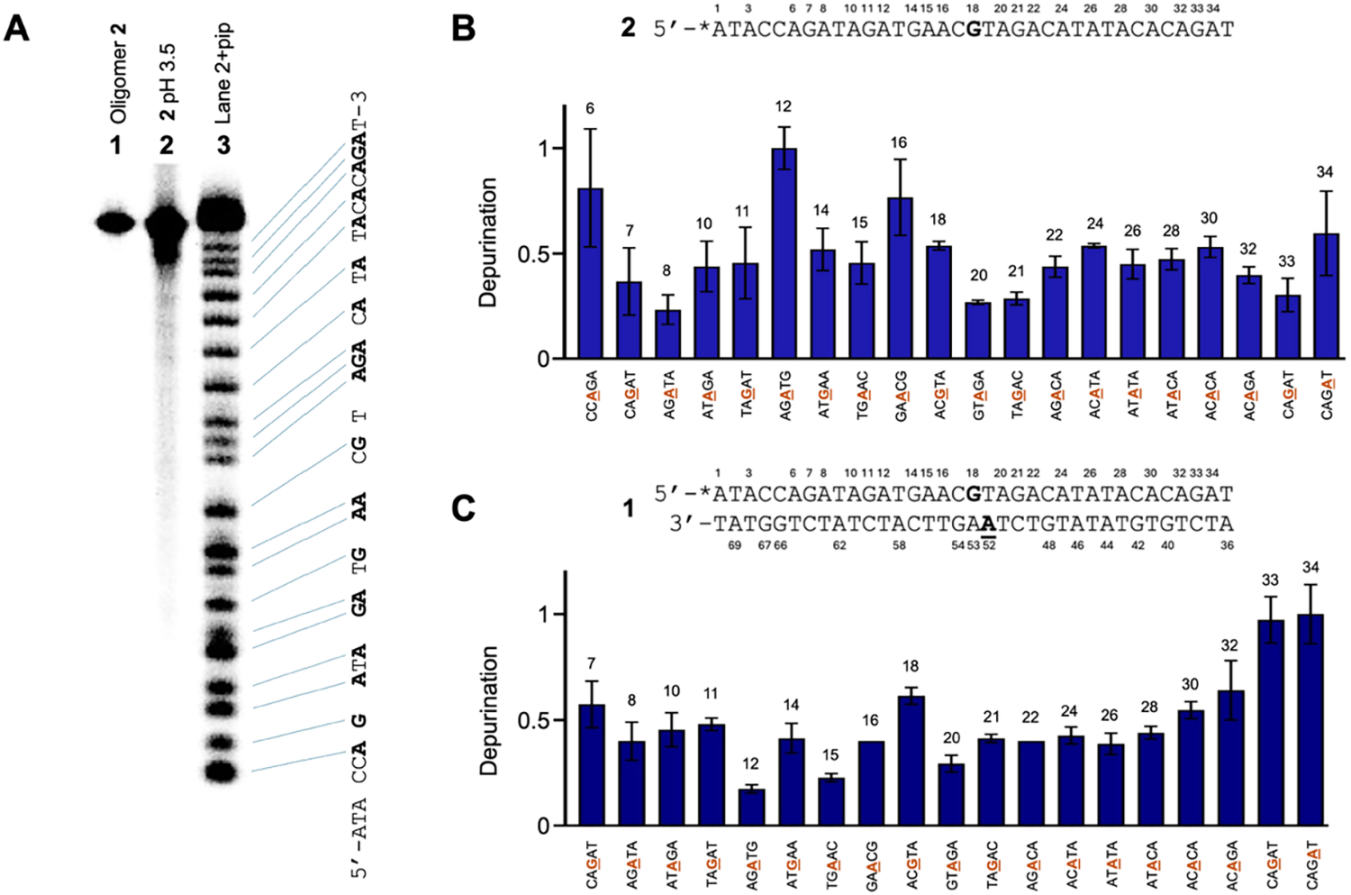
Gel electrophoretic measurement of depurination reactions in DNA duplex **1** and in the single-stranded DNA oligomer **2** at pH 3.5. (**Panel A)** Lane 1: 35 nucleotide 5′-Cy5-labeled DNA oligomer **2**. Lane 2: DNA oligomer **2** incubated in pH-3.5 citrate-phosphate buffer (17 mM and 33 mM, respectively, containing 100 mM NaCl) at 37 °C for 120 h (without base workup). Lane 3: DNA oligomer **2** incubated in pH-3.5 citrate-phosphate buffer (17 mM and 33 mM, respectively, containing 100 mM NaCl) at 37 °C for 120 h, followed by treatment with aqueous piperidine (1 M, 95 °C, 30 min) to convert AP sites into strand cleavage events. The DNA oligomers in the reactions were resolved by electrophoresis on a 0.4 mm thick 20% denaturing polyacrylamide gel at 500 V for 14 h and the Cy5-labeled fragments visualized by fluorescence imaging. Experiments were repeated at least three times. (**Panel B**) Bar graph representing the normalized depurination yield at each purine residue resolved in the gel electrophoretic analysis of DNA oligomer **2** (incubated in pH-3.5 buffer for 240 h). The depurination reaction in lane 3 of Panel A and represented in Panel B, with a total cleavage yield of 52%, is not considered single-hit conditions and, as a result, may lead to some “over-counting” of smaller cleavage fragments. (**Panel C**) Bar graph representing the normalized depurination yield at each purine residue resolved in the gel electrophoretic analysis of DNA duplex **1** (incubated in pH-3.5 buffer for 240 h).

We next examined depurination in duplex **1** incubated in pH 3.5 buffer at 37 °C for 240 h. The total yield of depurination events in duplex **1** across all sites resolved in the gel was 23%, with a 240 h reaction time, compared to 52% in the single-stranded DNA oligomer **2**, with a 120 h reaction time. These results are generally consistent with published results showing that depurination is approximately 3-fold slower in duplex DNA compared to single-stranded DNA.^9,11^ The locations of depurination hotspots in duplex **1** were different than those observed in single-stranded DNA oligomer **2**, with depurination seen at all A and G residues, but without any clear preference for loss of either A or G residues (Figures 2C and S2). Overall, these experiments showed that acidic conditions induced depurination, at least to some extent, at all A and G residues in the single-stranded DNA oligomer **2** and the DNA duplex **1**. The results further provided a map of the depurination hotspots in the labeled strand of duplex **1**.

### Acid-induced ICL Formation in Duplex **1**

We used gel electrophoretic analysis to examine whether conditions of acid stress (pH 3.5 and 37 °C) can induce ICLs in DNA duplex **1** via a multistep process involving acid-catalyzed depurination followed by reaction of the resulting AP site with purine residues on the opposing strand. At the outset, we generated authentic gel electrophoretic markers for two known types of AP-derived ICLs, using our published procedures (duplexes **5** and **6**; Figures 1 and S1).^17,31^ Briefly, the authentic AP-containing duplex **4** (Figure 1) was prepared by treatment of the 2′-deoxyuridine-containing precursor duplex **3** with the enzyme uracil DNA glycosylase (UDG).^53–55^ NaOH-induced strand cleavage was used to verify successful generation of the AP site (Figure 3, lanes 2 and 3).^29^ Incubation of the AP-containing duplex **4** in pH 7.4 buffer produced an authentic standard for the full-length, cross-linked duplex **5** containing the dA-AP linkage (Figure 3, lane 7).^17^ An authentic standard for duplex **6** containing the dA-PUA ICL, derived from strand cleavage at the AP site (Figures 1, S1 and S3) was prepared by incubation of duplex **4** in pH 7.4 buffer containing the low molecular weight AP lyase, spermine.^29–31^ Duplexes containing these AP-derived cross-links display slow gel mobility relative to the full-length, single-stranded, AP-containing oligomer,^16,17^ appearing near the top of the gel images shown here (Figure 3).

**Figure 3:**
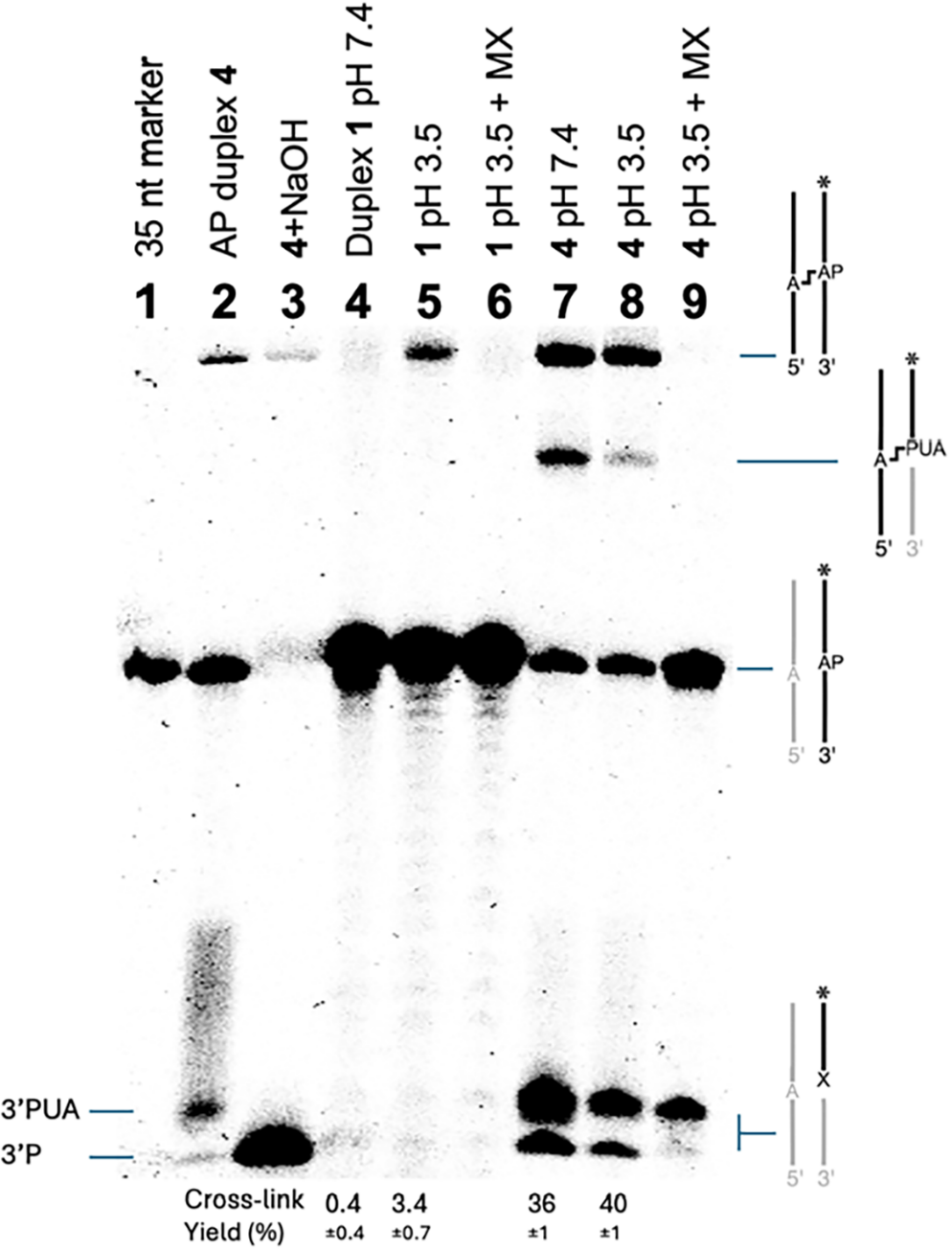
Gel electrophoretic evidence for the formation of AP-derived ICLs in duplex **1** at pH 3.5. Lane 1: 35 nt 5′-Cy5-labeled dU duplex **3**. Lane 2: A marker lane, containing the authentic, enzymatically generated AP-containing duplex **4**. Lane 3: AP-containing duplex **4** treated with NaOH (200 mM at 60 °C for 30 min) to induce strand cleavage at the AP site. Lane 4: Duplex **1** incubated in pH 7.4 HEPES buffer (50 mM, containing 100 mM NaCl) at 37 °C for 120 h. Lane 5: Duplex **1** incubated in pH 3.5 citrate-phosphate buffer (17 mM and 33 mM, respectively, containing 100 mM NaCl) at 37 °C for 120 h. Lane 6: Duplex **1** incubated with methoxyamine (2 mM) in pH 3.5 citrate-phosphate buffer (17 mM and 33 mM, respectively, containing 100 mM NaCl) at 37 °C for 120 h. Lane 7: A control reaction in which duplex **4**, containing the authentic enzymatically generated AP site, was incubated in pH 7.4 HEPES buffer (50 mM, containing 100 mM NaCl) at 37 °C for 120 h to generate the authentic dA-AP ICL. The faster-migrating strand cleavage products observed in the reactions of **4** result from handling of the AP-containing duplex during the sample handling. Lane 8: A control reaction in which duplex **4**, containing the authentic enzymatically generated AP site, was incubated in pH 3.5 citrate-phosphate buffer (17 mM and 33 mM, respectively, containing 100 mM NaCl) at 37 °C for 120 h to generate the dA-AP ICL. Lane 9: the AP-containing duplex **4** incubated with methoxyamine (5 mM) in pH-3.5 citrate-phosphate buffer (17 mM and 33 mM, respectively, containing 100 mM NaCl) at 37 °C for 120 h. The DNA oligomers in the reactions were resolved by electrophoresis on a 0.4 mm thick 20% denaturing polyacrylamide gel at 500 V for 14 h and the Cy5-labeled fragments visualized by fluorescence imaging. Experiments were repeated at least three times. Chemical structures for the dA-AP and dA-PUA cross-links are shown in Scheme 1 and Figure S1. In the cartoons shown on the right side of the gel, the asterisk (*) denotes a Cy5 fluorescent label. The labeled fragments, shown in black, are observed in the gel. The unlabeled DNA fragments shown in gray are not visible in the gel. The X indicates a 3′-phosphoryl, -deoxyribose, or -PUA end group.

To investigate whether depurination events in duplex **1** lead to ICL formation under conditions of acid stress, we incubated duplex **1** in pH 3.5 citrate-phosphate buffer at 37 °C. Gel electrophoretic analysis revealed the formation of a slowly migrating band in 3.4 ± 0.7% yield (Figure 3, lane 5). The slowly migrating product generated by incubation of duplex **1** under acidic conditions displayed the same gel mobility as the authentic, full-length cross-linked duplex **5** (Figure 3, lanes 7 and 8, and Figure S3). Acid-induced cross-link formation in duplex **1** was inhibited by the presence of methoxyamine (CH_3_ONH_2_, MX) in the reaction mixture (Figure 3, lanes 5 versus 6). This provided evidence that the slowly migrating band was an AP-derived reaction product, because MX captures AP sites as an inert oxime derivative.^17,60^ Incubation of duplex **1** in pH 7.4 buffer, a condition where depurination yields are low,^9,11^ produced little or no cross-linked DNA (Figure 3, lane 4). Overall, the results were consistent with the generation of an AP-derived cross-link(s) in duplex **1** under conditions of acid stress.

### Salt and pH Effects on Acid-Induced ICL Formation in Duplex **1**

Cells contain significant concentrations of sodium, potassium, and magnesium ions. To examine whether salt affects the formation of AP-derived ICLs under conditions of acid stress, we varied sodium ion (50–150 mM), potassium ion (20 mM), and magnesium ion (10 mM) concentrations in citrate-phosphate buffer (pH 3.5). We observed no significant salt effects on acid-induced ICL formation in duplex **1** (data not shown).

We also examined the yields of ICL formation in duplex **1** across a range of pH values (7.4, 5.0, 4.0, 3.5, 3.0, and 2.5). The highest ICL yield was observed at pH 3.5 (Figures 4 and S4). ICL formation was also observed at pH 4. This result may be significant because *E. coli* strains with functional acid resistance systems can experience intracellular pH values near 4 when exposed to conditions of extreme acid stress (pH 2.5).^57,61^

**Figure 4:**
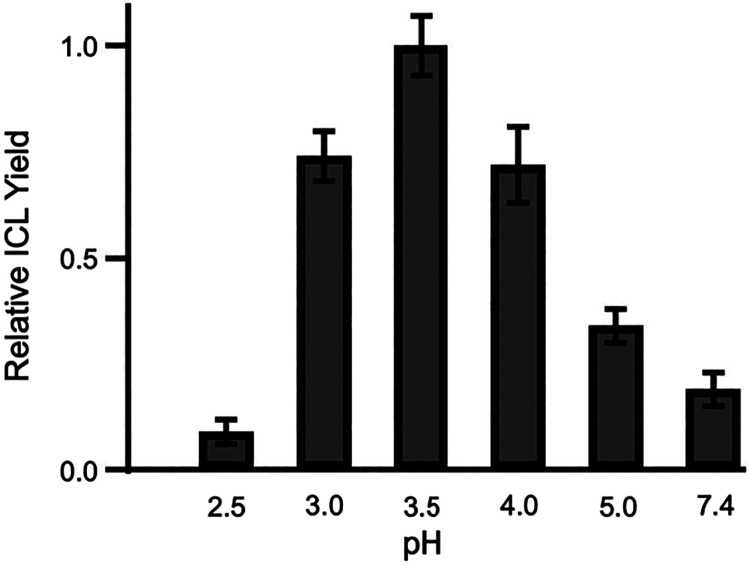
Effect of pH on the yield of ICL formation in duplex **1**. Duplex **1** was incubated in citrate-phosphate buffers at the indicated pH values (17 mM and 33 mM, respectively, containing 100 mM NaCl) at 37 °C for 120 h.

The pH optimum of 3.5 observed for ICL formation must reflect an interplay of pH effects on the depurination reaction and the cross-linking process. Depurination rates increase almost linearly across the pH range from 7 to 1.^11^ On the other hand, the equilibrium yields of AP-derived cross-links do not vary substantially across pH values of 5–7.^56^

In the current work, the low ICL yields observed above pH 3.5 almost certainly reflect low depurination yields at higher pH values. The low ICL yields seen below pH 3.5 may reflect the inhibitory effects of low pH on the imine formation step of the cross-linking process.^62^ In addition, low ICL yields below pH 3.5 may result from destabilization of the DNA duplex. Low pH destabilizes duplex DNA by protonation of cytosine residues.^63,64^ With this in mind, it may be important to emphasize that two of our results provide evidence that duplexes **1** and **4** remain hybridized at pH 3.5. First, the observed ICL formation in these duplexes provides evidence that they exist in the double-stranded form, given that the formation of AP-derived ICLs is known to require DNA hybridization.^56^ Indeed, in duplex **4**, containing an authentic, enzymatically generated AP site, the ICL yield at pH 3.5 was slightly *greater* than that obtained in pH 7.4 buffer Figure 3, lanes 7 and 8). Second, in experiments at pH 3.5, we observed the characteristic^9,11^ decrease in the yield of acid-catalyzed depurination in duplex DNA (**1**) compared to that in the single-stranded DNA oligomer (**2**, Figure 2).

Published literature also provides evidence that DNA duplexes can exist in pH-3.5 buffer. For example, a significant fraction of the self-complementary, 10-nucleotide DNA oligomer TACGCGCGTA exists in the hybridized form at pH 3.5 and 37 °C (in 20 mM sodium phosphate buffer containing 16 mM NaCl).^65^ The higher salt concentration used in our experiments (100 mM NaCl) is expected to further stabilize the duplex form.^63,65^ Single-molecule stretching experiments also provide evidence that larger DNA fragments remain double-stranded at pH 3.5.^66^ Overall, it seems clear that our DNA duplexes remain hybridized at pH 3.5, in the experiments described here. On the other hand, it is reasonable to suspect that the low ICL yields observed at pH values of 2.5 and 3.0 could be, at least in part, a consequence of duplex destabilization.

### Position G18 Is a Major Site for Acid-Induced ICL Formation in Duplex **1**

There are 36 purine residues in duplex **1**. Of these, 25 reside in sequence environments that confer the potential for AP-derived ICL formation (annotated by arrows in Figure S1). ICL formation at each of these sites was not expected to be equal, both because the amount of depurination at each site is not equal (Figure 2) and because the yield of AP-derived ICLs is dependent on local sequence.^16,56^ We anticipated that the AP site generated by depurination of G18 in duplex **1** might be a preferred site for ICL formation. This expectation was based on previous work showing that an AP site located in this sequence generates a high yield of the dA-AP ICL (60%, 5′ACXTA/TAAGT, where X = AP, where the ICL attachment is at the mispaired A indicated by the underline).^17,56,67^ The expectation that ICL formation might be favored at position 18 was reinforced by our results showing that G18 is a depurination hotspot site in duplex **1** (Figure 2C).

To test whether depurination and ICL formation at position 18 were responsible for substantial amounts of acid-induced cross-link formation in duplex **1**, we compared the cross-link yield formed in duplex **1** with that generated in duplex **7**, where the critical guanine residue at position 18 was replaced by a thymine residue. As a result of this sequence permutation, duplex **7** lacks the capacity to form the critical AP site at position 18 under acidic conditions. In the event, we found that incubation of duplex **7** under conditions of acid stress gave a 4.5-fold lower ICL yield compared to duplex **1** (0.9% versus 3.4%, Figure 5). This result indicated that the formation of the AP site at position 18 played an important role in the cross-linking of duplex **1** under conditions of acid stress. At the same time, the results provided evidence that ICLs are generated in duplex **7** at locations other than position 18 (Figures 5 and S5).

**Figure 5:**
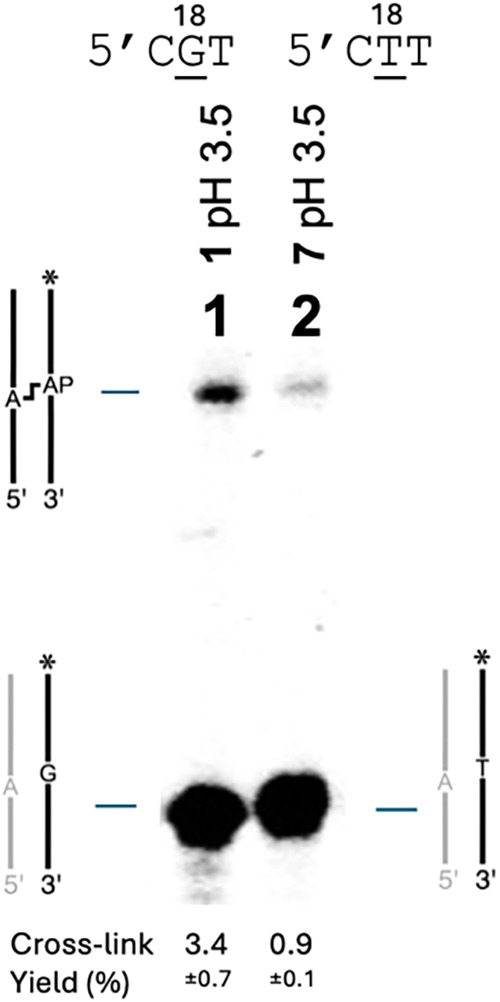
Position 18 In Duplex **1** Is a Major Site for Acid-Induced ICL Formation. The yield of acid-induced ICL formation in duplex **7**, lacking the critical G residue at position 18, is low compared to the ICL yield in duplex **1** at pH 3.5. Experiments were repeated at least three times.

## Conclusions

Our findings demonstrate that ICLs can be generated under conditions of acid stress by a sequential process involving acid-catalyzed depurination, followed by reaction of the resulting AP site with an adenine residue on the opposing strand of duplex DNA. Our results are consistent with very early work by Freese and Cashel, who provided evidence that exposure of *Bacillus subtilis* DNA to low pH (3.75–5) led to the formation of some (at that time structurally uncharacterized) AP-derived ICLs.^79^

While the yield of AP-derived ICLs may be low, these lesions can have outsized biological consequences because the covalent connection of the opposing strands in duplex DNA completely blocks the read-out and replication of the genetic information and requires complex, multistep processes for repair.^12,50,68^ Indeed, it has been suggested that a single ICL may be sufficient to kill a bacterial cell.^69^ Overall, the results support the possibility that the formation of ICLs derived from AP sites could be a genotoxic consequence of acid stress in bacterial cells. It remains uncertain whether AP-derived cross-links are substrates for the acid resistance protein AlkX from *Acinetobacter baumannii*.^7^ It is also possible that oxidative stress arising as a consequence of acid stress in bacteria^70^ generates other types of ICLs.^50,71–74,80^

Finally, the acid-induced generation of ICLs examined in this work could be important in a variety of contexts in addition to bacterial acid stress, including thymineless cell death,^3^ the degradation of environmental DNA, DNA-based data storage systems, ancient DNA, and DNA-encoded libraries.^75–78^

## Supplementary Material



## Notes

Representative chemical structures of ICLs generated by AP sites in duplex DNA, gel electrophoretic analysis of depurination in duplex **1** under acidic conditions, generation of a gel electrophoretic marker for an ICL derived from strand cleavage at an AP site in DNA, gel electrophoretic data measuring pH effects on ICL formation in duplex **1** (PDF)

## References

[1] Guan N., Liu L. (2020). Microbial response to acid stress: mechanisms and applications. Appl. Microbiol. Biotechnol..

[2] Schumacher K., Brameyer S., Jung K. (2023). Bacterial
acid stress response: from
cellular changes to antibiotic tolerance and phenotypic heterogeneity. Curr. Opin. Microbiol..

[3] Ketcham A., Freddolino P. L., Tavazoie S. (2022). Intracellular acidification
is a
hallmark of thymineless death in *E. coli*. PLoS Genet..

[4] Zheng Y., Wang J., Bai X., Chang Y., Mou J., Song J., Wang M. (2018). Improving
the acetic acid tolerance
and fermentation of Acetobacter pasteurianus by nucleotide excision
repair protein UvrA. Appl. Microbiol. Biotechnol..

[5] Jeong K. C., Hung K. F., Baumler D. J., Byrd J. J., Kaspar C. W. (2008). Acid stress
damage of DNA is prevented by Dps binding in *Escherichia
coli* O157:H7. BMC Microbiol..

[6] de
la Garza-García J. A., Ouahrani-Bettache S., Lyonnais S., Ornelas-Eusebio E., Freddi L., Al Dahouk S., Occhialini A., Kohler S. (2021). Comparative Genome-Wide Transcriptome
Analysis of Brucella suis and Brucella microti Under Acid Stress at
pH 4.5: Cold Shock Protein CspA and Dps Are Associated With Acid Resistance
of B. microti. Front. Microbiol..

[7] Kunkle D. E., Cai Y., Eichman B. F., Skaar E. P. (2024). An interstrand DNA crosslink glycosylase
aids Acinetobacter baumannii pathogenesis. Proc.
Natl. Acad. Sci. U. S. A..

[8] Lindahl T., Karlstrom O. (1973). Heat-induced depyrimidination of deoxyribonucleic acid
in solution. Biochemistry.

[9] Lindahl T., Nyberg B. (1972). Rate of depurination
of native deoxyribonucleic acid. Biochemistry.

[10] Gates K. S. (2009). An overview
of chemical processes that damage cellular DNA: spontaneous hydrolysis,
alkylation, and reactions with radicals. Chem.
Res. Toxicol..

[11] An R., Jia Y., Wan B., Zhang Y., Dong P., Li J., Liang X. (2014). Non-enzymatic depurination of nucleic acids: factors and mechanisms. PLoS One.

[12] Price N. E., Gates K. S. (2024). Novel processes
associated with the repair of interstrand
cross-links derived from abasic sites in duplex DNA: roles for the
base excision repair glycosylase NEIL3 and the SRAP protein HMCES. Chem. Res. Toxicol..

[13] Thompson P. S., Cortez D. (2020). New insights into abasic
site repair and tolerance. DNA Repair.

[14] Schaaper R. M., Kunkel T. A., Loeb L. A. (1983). Infidelity
of DNA synthesis associated
with bypass of apurinic sites. Proc. Natl. Acad.
Sci. U. S. A..

[15] Choi J.-Y., Lim S., Kim E. J., Jo A., Guengerich F. P. (2010). Translesion
synthesis across abasic lesions by human B-family and Y-family DNA
polymerases alpha, delta, eta, iota, kappa, and REV. J. Mol. Biol..

[16] Johnson K. M., Price N. E., Wang J., Fekry M. I., Dutta S., Seiner D. R., Wang Y., Gates K. S. (2013). On the Formation
and Properties of Interstrand DNA-DNA Cross-links Forged by Reaction
of an Abasic Site With the Opposing Guanine Residue of 5′-CAp
Sequences in Duplex DNA. J. Am. Chem. Soc..

[17] Price N. E., Johnson K. M., Wang J., Fekry M. I., Wang Y., Gates K. S. (2014). Interstrand DNA–DNA
Cross-Link Formation Between
Adenine Residues and Abasic Sites in Duplex DNA. J. Am. Chem. Soc..

[18] Varela J. G., Pierce L. E., Guo X., Price N. E., Johnson K. M., Yang Z., Wang Y., Gates K. S. (2021). Interstrand Cross-Link
Formation Involving Reaction of a Mispaired Cytosine Residue with
an Abasic Site in Duplex DNA. Chem. Res. Toxicol..

[19] Nejad M. I., Price N. E., Haldar T., Lewis C., Wang Y., Gates K. S. (2019). Interstrand DNA
cross-links derived from reaction of
a 2-aminopurine residue with an abasic site. ACS Chem. Biol..

[20] Gamboa
Varela J., Gates K. S. (2015). A Simple, High-Yield Synthesis of
DNA Duplexes Containing a Covalent, Thermally-Reversible Interstrand
Cross-link At a Defined Location. Angew. Chem.,
Int. Ed. Engl..

[21] Bryan C., Cepeda J., Li B., Yang K. (2025). DNA-Protein Cross-Links
Derived from Abasic DNA Lesions: Recent Progress and Future Directions. Chem. Res. Toxicol..

[22] Bryan C., Le J., Wei X., Yang K. (2023). Saccharomyces cerevisiae apurinic/apyrimidinic
endonuclease 1 repairs abasic site-mediated DNA-peptide/protein cross-links. DNA Repair (Amst).

[23] Bryan C., Wei X., Wang Z., Yang K. (2022). In vitro eradication
of abasic site-mediated
DNA-peptide/protein cross-links by *Escherichia coli* long-patch base excision repair. J. Biol.
Chem..

[24] Yudkina A. V., Bulgakov N. A., Kim D. V., Baranova S. V., Ishchenko A. A., Saparbaev M. K., Koval V. V., Zharkov D. O. (2023). Abasic
site-peptide
cross-links are blocking lesions repaired by AP endonucleases. Nucleic Acids Res..

[25] Marchand C., Krajewski K., Lee H.-F., Antony S., Johnson A. A., Amin R., Roller P. P., Kvaratskhelia M., Pommier Y. (2006). Covalent binding of
the natural antimicrobial peptide
indolicidin to DNA abasic sites. Nucleic Acids
Res..

[26] Gomina A., Islam T., Shim G., Lei Z., Gates K. S. (2024). Reactions
of DNA abasic sites with 1,2-aminothiols including cysteamine and
peptides containing N-terminal cysteine residues. Chem. Res. Toxicol..

[27] Tang J., Tang F., Zhao L. (2022). Facile preparation
of model DNA interstrand
cross-link repair intermediates using ribonucleotide-containing DNA. DNA Repair.

[28] Lindahl T., Andersson A. (1972). Rate of chain breakage at apurinic
sites in double-stranded
deoxyribonucleic acid. Biochemistry.

[29] Haldar T., Jha J. S., Yang Z., Nel C., Housh K., Cassidy O. J., Gates K. S. (2022). Unexpected Complexity
in the Products
Arising from NaOH-, Heat-, Amine-, and Glycosylase-Induced Strand
Cleavage at an Abasic Site in DNA. Chem. Res.
Toxicol..

[30] Housh K., Jha J. S., Yang Z., Haldar T., Johnson K. M., Yin J., Wang Y., Gates K. S. (2021). Formation and Repair of an Interstrand
DNA Cross-Link Arising from a Common Endogenous Lesion. J. Am. Chem. Soc..

[31] Yang Z., Price N. E., Johnson K. M., Wang Y., Gates K. S. (2017). Interstrand
cross-links arising from strand breaks at true abasic sites in duplex
DNA. Nucleic Acids Res..

[32] Chen Y. H., Esparza Sanchez M., Sanchez M. E., Hung T. I., Tang J., Xu W., Yin J., Wang Y., Chang C.-E. A., Zhang H., Chen J. (2025). Glutathionylated
DNA Adducts Accumulate in Mitochondrial DNA and
Are Regulated by AP Endonuclease 1 and Tyrosyl-DNA Phosphodiesterase
1. Proc. Natl. Acad. Sci. U.S.A..

[33] Jha J. S., Yin J., Haldar T., Yang Z., Wang Y., Gates K. S. (2022). Reconsidering
the Chemical Nature of Strand Breaks Derived from Abasic Sites in
Cellular DNA: Evidence for 3′-Glutathionylation. J. Am. Chem. Soc..

[34] Yin J., Gates K. S., Wang Y. (2022). N-Methyl-N-nitrosourea
Induced 3′-Glutathionylated
DNA-Cleavage Products in Mammalian Cells. Anal.
Chem..

[35] Bryan C., Yang K. (2025). Human 8-oxoguanine glycosylase OGG1
cleaves abasic sites and covalently
conjugates to 3′-DNA termini via cysteine and histidine addition. ChemBioChem..

[36] Wei X., Yang K. (2023). PARP1 Incises
Abasic Sites and Covalently Cross-links to 3′-DNA
Termini via Cysteine Addition Not Reductive Amination. Biochemistry.

[37] Xu W., Tang J., Zhao L. (2023). DNA-protein
cross-links between abasic
DNA damage and mitochondrial transcription factor A (TFAM). Nucleic Acids Res..

[38] Ensminger M., Iloff L., Ebel C., Nikolova T., Kaina B., Lobrich M. (2014). DNA breaks and chromosomal aberrations arise when replication
meets base excision repair. J. Cell Biol..

[39] Kuzminov A. (2001). Single-strand
interruptions in replicating chromosomes cause double-strand breaks. Proc. Natl. Acad. Sci. U. S. A..

[40] Hanthi Y. W., Ramirez-Otero M. A., Appleby R., De Antoni A., Joudeh L., Sannino V., Waked S., Ardizzoia A., Barra V., Fachinetti D. (2024). RAD51 protects abasic
sites to prevent replication fork breakage. Mol. Cell.

[41] Kunkel T. A. (1984). Mutational
specitificity of depurination. Proc. Natl. Acad.
Sci. U.S.A..

[42] Schaaper R. M., Loeb L. A. (1981). Depurination causes mutagenesis in SOS-induced cells. Proc. Natl. Acad. Sci. U.S.A..

[43] Bradley N. P., Washburn L. A., Christov P. P., Watanabe C. M. H., Eichman B. F. (2020). *Escherichia coli* YcaQ is a DNA glycosylase that unhooks
DNA interstrand crosslinks. Nucleic Acids Res..

[44] Imani-Nejad M., Housh K., Rodriguez A. A., Haldar T., Kathe S., Wallace S. S., Eichman B. F., Gates K. S. (2020). Unhooking of an
interstrand cross-link at DNA fork structures by the DNA glycosylase
NEIL3. DNA Repair.

[45] Mullins E. A., Warren G. M., Bradley N. P., Eichman B. F. (2017). Structure of a DNA
glycosylase that unhooks interstrand cross-links. Proc. Natl. Acad. Sci. U.S.A..

[46] Chen X., Sun Y., Wang S., Ying K., Xiao L., Liu K., Zuo X., He J. (2020). Identification of a novel structure-specific endonuclease
AziN that contributes to the repair of azinomycin B-mediated DNA interstrand
crosslinks. Nucleic Acids Res..

[47] Oswalt L. E., Eichman B. F. (2024). NEIL3: A unique
DNA glycosylase involved in interstrand
DNA crosslink repair. DNA Repair.

[48] Grossmann K. F., Ward A. M., Matkovic M. E., Folias A. E., Moses R. E. (2001). S. cerevisiae
has three pathways for DNA interstrand crosslink repair. Mutat. Res..

[49] Reddy M. C., Vasquez K. M. (2005). Repair of genome
destabilizing lesions. Radiat. Res..

[50] Housh K., Jha J. S., Haldar T., Binth Md Amin S., Islam T., Wallace A., Gomina A., Guo X., Nel C., Wyatt J. W., Gates K. S. (2021). Formation and repair
of unavoidable,
endogenous interstrand cross-links in cellular DNA. DNA Repair.

[51] McIlvaine T. C. (1921). A buffer
solution for colorimetric comparison. J. Biol.
Chem..

[52] Sambrook, J. ; Fritsch, E. F. ; Maniatis, T. Molecular Cloning: A Lab Manual; Cold Spring Harbor Press, 1989.

[53] Varshney U., van de Sande J. H. (1991). Specificities
and kinetics of uracil excision from
uracil-containing DNA oligomers by *Escherichia coli* uracil DNA glycosylase. Biochemistry.

[54] Stuart G. R., Chambers R. W. (1987). Synthesis and properties
of oligodeoxynucleotides with
an AP site at a preselected position. Nucleic
Acids Res..

[55] Lindahl T., Ljunquist S., Siegert W., Nyberg B., Sperens B. (1977). DNA N-glycosidases:
properties of uracil-DNA glycosidase from *Escherichia
coli*. J. Biol. Chem..

[56] Amin S. B. M., Islam T., Price N. E., Wallace A., Guo X., Gomina A., Heidari M., Johnson K. M., Lewis C. D., Yang Z., Gates K. S. (2022). Effects
of local sequence, reaction
conditions, and various additives on the formation and stability of
interstrand cross-links derived from the reaction of an abasic site
with an adenine residue in duplex DNA. ACS Omega.

[57] Richard H., Foster J. W. (2004). *Escherichia
coli* glutamate-
and arginine-dependent acid resistance systems increase internal pH
and reverse transmembrane potential. J. Bacteriol..

[58] Lund P., Tramonti A., De Biase D. (2014). Coping with low pH: molecular strategies
in neutralophilic bacteria. FEMS Microbiol Rev..

[59] Giusti W. G., Adriano T. (1993). Synthesis and characterization of 5′-fluorescent-dye-labeled
oligonucleotides. Genome Res..

[60] Rosa S., Fortini P., Karran P., Bignami M., Dogliotti E. (1991). Processing
in vitro of an abasic site reacted with methoxyamine: a new assay
for the detection of abasic sites formed in vivo. Nucleic Acids Res..

[61] Yang M., Jalloh A. S., Wei W., Zhao J., Wu P., Chen P. R. (2014). Biocompatible click
chemistry enabled compartment-specific
pH measurement inside *E. coli*. Nat. Commun..

[62] Cordes E. H., Jencks W. P. (1962). On the mechanism of Schiff base formation and hydrolysis. J. Am. Chem. Soc..

[63] Zimmer C., Venner H. (1966). Protonation of cytosine in DNA. Biopolymers.

[64] González-Olvera J. C., Durec M., Marek R., Fiala R., Morales-Garcia M., Gonzalez-Jasso E., Pless R. C. (2018). Protonation of Nucleobases in Single-
and Double-Stranded DNA. Chembiochem.

[65] Ashwood B., Sanstead P. J., Dai Q., He C., Tokmakoff A. (2020). 5-Carboxylcytosine
and Cytosine Protonation Distinctly Alter the Stability and Dehybridization
Dynamics of the DNA Duplex. J. Phys. Chem. B.

[66] Williams M. C., Wenner J. R., Rouzina I., Bloomfield V. A. (2001). Effect
of pH on the overstretching transition of double-stranded DNA: evidence
of force-induced DNA melting. Biophys. J..

[67] Kellum A. H., Qiu D. Y., Voehler M. W., Martin W., Gates K. S., Stone M. P. (2021). Structure of a Stable Interstrand DNA Cross-Link Involving
a β-N-Glycosyl Linkage Between an N6-dA Amino Group and an Abasic
Site. Biochemistry.

[68] Semlow D. R., Zhang J., Budzowska M., Drohat A. C., Walter J. C. (2016). Replication-dependent
unhooking of DNA interstrand cross-links by the NEIL3 glycosylase. Cell.

[69] Lawley P. D., Phillips D. H. (1996). DNA adducts from chemotherapeutic agents. Mutat. Res..

[70] Maurer L. M., Yohannes E., Bondurant S. S., Radmacher M., Slonczewski J. L. (2005). pH regulates genes for flagellar
motility, catabolism,
and oxidative stress in *Escherichia coli* K-12. J. Bacteriol..

[71] Wang J., Takyi N. A., Hsiao Y. C., Tang Q., Chen Y. T., Liu C. W., Ma J., Qi R., Bian K., Peng Z. (2024). Stable Interstrand Cross-Links
Generated from the Repair
of 1,N(6)-Ethenoadenine in DNA by alpha-Ketoglutarate/Fe­(II)-Dependent
Dioxygenase ALKBH2. J. Am. Chem. Soc..

[72] Rozelle A. L., Cheun Y., Vilas C. K., Koag M. C., Lee S. (2021). DNA interstrand
cross-links induced by the major oxidative adenine lesion 7,8-dihydro-8-oxoadenine. Nat. Commun..

[73] Rozelle A. L., Zhang L. B., Lee S. (2025). Effect of single strand breaks on
the formation of DNA interstrand crosslinks induced by the major oxidative
adenine lesion 7,8-dihydro-8-oxoadenine. Nucleosides,
Nucleotides Nucleic Acids.

[74] Stone M. P., Cho Y. J., Huang H., Kim H. Y., Kozekov I. D., Kozekova A., Wang H., Minko I. G., Lloyd R. S., Harris T. M. (2008). Interstrand
cross-links induced by alpha, beta-unsaturated
aldehydes derived from lipid peroxidation and environmental sources. Acc. Chem. Res..

[75] Strickler K. M., Fremier A. K., Goldberg C. S. (2015). Quantifying effects of UV-B, temperature,
and pH and eDNA degradation in aquatic microcosms. Biol. Conservation.

[76] Seymour M., Durance I., Cosby B. J., Ransom-Jones E., Deiner K., Ormerod S. J., Colbourne J. K., Wilgar G., Carvalho G. R., de Bruyn M. (2018). Acidity
promotes degradation of multi-species environmental DNA in lotic mesocosms. Commun. Biol..

[77] Dabney J., Meyer M., Pääbo S. (2013). Ancient DNA
damage. Cold Spring Harb. Perspect. Biol..

[78] Sunkari Y. K., Nguyen T. L., Siripuram V. K., Flajolet M. (2023). Impact of organic chemistry
conditions on DNA durability in the context of DNA-encoded library
technology. iScience.

[79] Freese E., Cashel M. (1964). Crosslinking of deoxyribonucleic
acid by exposure to
low pH. Biochim. Biophys. Acta, Spec. Sect.
Nucleic Acids Relat. Subj..

[80] Sczepanski J. T., Jacobs A. C., Majumdar A., Greenberg M. M. (2009). Scope and
Mechanism of Interstrand Cross-Link Formation by the C4′-Oxidized
Abasic Site. J. Am. Chem. Soc..

