# Supplementary material for: Generation of Interstrand DNA Cross-Links under Conditions of Acid Stress
Source: Chem Res Toxicol. 2026 Jun 5;39(7):1431–8. doi: 10.1021/acs.chemrestox.6c00217 (PMC13390037; doi:10.1021/acs.chemrestox.6c00217)
Supplement: Supplementary file 1 [file tx6c00217_si_001.pdf]

Supporting Information for

Generation of Interstrand DNA Cross-links Under Conditions of Acid Stress

Mithila Farzana Mumu,[a] Marjan Heidari,[a] Md. Selim Mahbub,[a] and Kent S. Gates[a,b,*]

[a]Department of Chemistry, 125 Chemistry Bldg,
University of Missouri, Columbia, MO 65211, United States

[b]Department of Biochemistry, 125 Chemistry Bldg,
University of Missouri, Columbia, MO 65211, United States

*E-mail: gatesk@missouri.edu; Tel: (573) 882-6763

Table of Contents

Figure S1. Representative structures of ICLs derived from AP sites in duplex DNA and potential locations for the formation of AP-derived ICLs in duplex **1**……………………………………...S1

Figure S2. Gel electrophoretic analysis of acid-catalyzed depurination in DNA duplex **1**……….S2

Figure S3. The full-size ICL generated by incubation of duplex **1** in pH 3.5 buffer migrates more slowly in a denaturing polyacrylamide gel than the cross-linked product derived from cleavage of an AP site in duplex **4**……………………………………………………….........................…S3

Figure S4. Gel electrophoretic analysis of ICL formation in DNA duplex **1** at different pH values.………………………………………………………………………………………S4

Figure S5. Complete gel image corresponding to Figure 5 in the main manuscript……………...S5

Figure S1

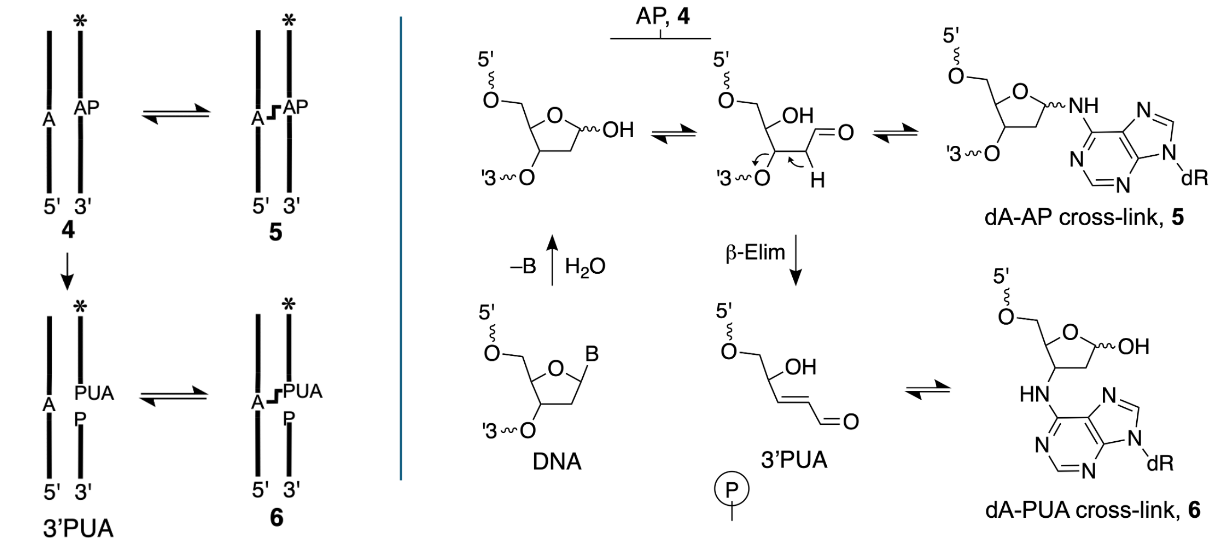

1 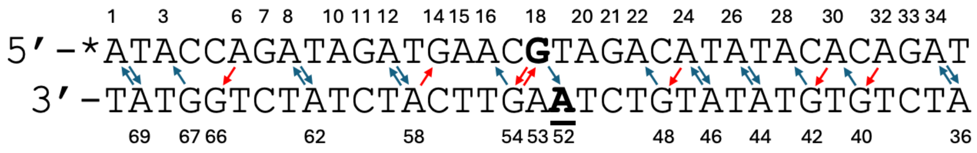

Figure S1. Representative structures of ICLs derived from AP sites in duplex DNA and potential locations for the formation of AP-derived ICLs in duplex **1**. Full-length ICLs such as **5** are derived from reaction of a nucleobase on the opposing strand with an AP site (Price, N. E.; Johnson, K. M.; Wang, J.; Fekry, M. I.; Wang, Y.; Gates, K. S. *J. Am. Chem. Soc*. **2014**, *136*, 3483-3490 and Johnson, K. M.; Price, N. E.; Wang, J.; Fekry, M. I.; Dutta, S.; Seiner, D. R.; Wang, Y.; Gates, K. S. *J. Am. Chem. Soc*. **2013**, *135,* 1015-1025). Duplexes containing an ICL adjacent to a strand break (**6**) are derived from reaction of an opposing nucleobase such as dA with the 3'PUA residue generated by β-elimination at an AP site in duplex DNA (Yang, Z.; Price, N. E.; Johnson, K. M.; Wang, Y.; Gates, K. S. *Nucleic Acids Res.* **2017**, 45, 6486-6493 and Housh, K.; Jha, J. S.; Yang, Z.; Haldar, T.; Johnson, K. M.; Yin, J.; Wang, Y.; Gates, K. S. *J. Am. Chem. Soc.* **2021**, *143*, 15344-15357). Here we abbreviate the PUA-derived cross-link as dA-PUA. In previous work, we referred to this cross-link as dA-ddR (ddR=2',3'-dideoxyribose). This is the cross-link abbreviated Y-A in Figure 1 of the main manuscript. In the illustration of duplex **1** above, the arrows represent sites with the capacity to generate AP-derived ICLs. The tail of each arrow signifies the location of an AP site generated by acid-induced depurination.

Figure S2

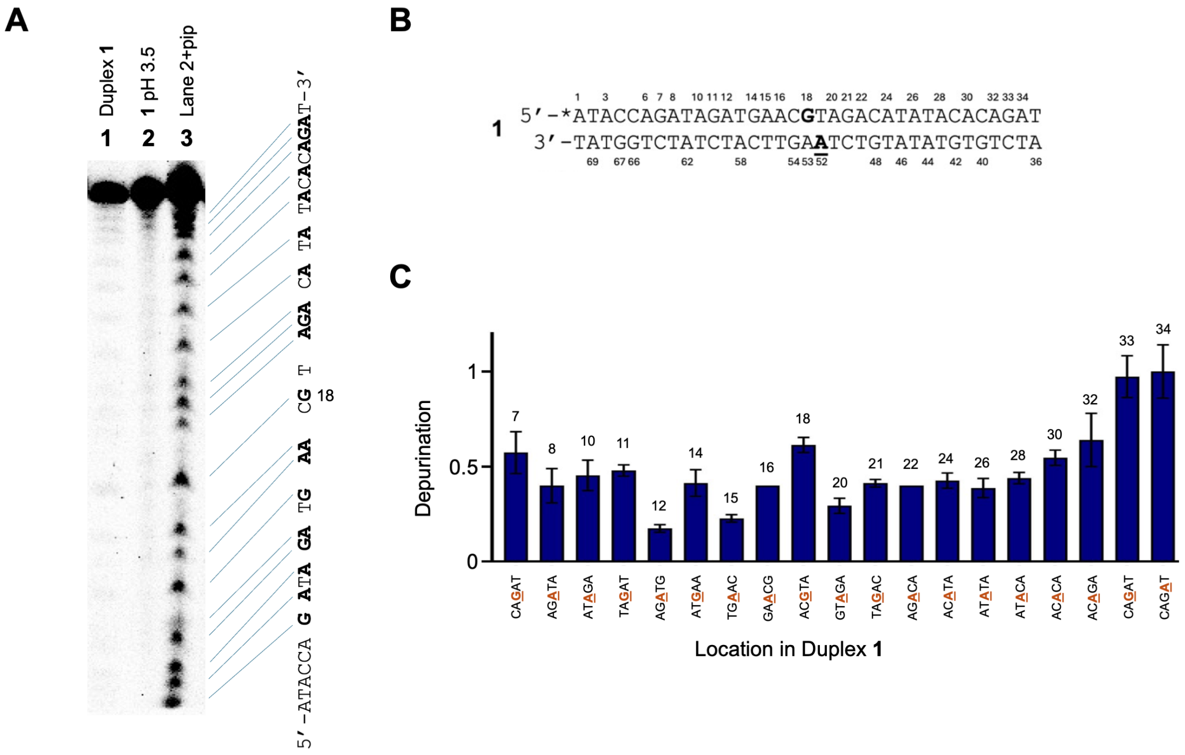

Figure S2. Gel electrophoretic analysis of acid-catalyzed depurination reactions in DNA duplex **1**. **Panel A**. Lane 1: 35 mer 5′-Cy5-labeled DNA duplex **1**. Lane 2: DNA duplex **1** incubated in pH 3.5 citrate-phosphate buffer (17 mM and 33 mM, respectively, containing 100 mM NaCl) at 37 ˚C for 240 h (without any base workup). Lane 3: DNA duplex **1** incubated in pH 3.5 citrate-phosphate buffer (17 mM and 33 mM, respectively, containing 100 mM NaCl) at 37 ˚C for 240 h, followed by treatment with piperidine (1 M, 95 ˚C, 30 min) to convert AP sites into strand cleavage events. The DNA oligomers in the reactions were resolved by electrophoresis on a 0.4 mm thick 20% denaturing polyacrylamide gel at 500 V for 14 h and the Cy5-labeled fragments visualized by fluorescence imaging. **Panel B**. The sequence of duplex **1**. **Panel C**. Bar graph representing the relative depurination yield at each purine residue resolved in the gel electrophoretic analysis of DNA duplex **1**.

Figure S3

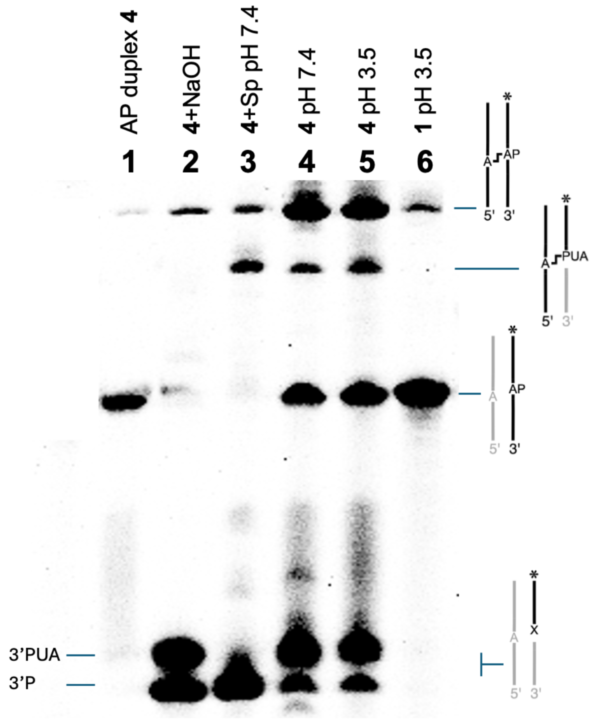

Figure S3. The cross-linked duplex generated under conditions of acid stress co-migrates with the authentic cross-linked duplex **5** and *not* the cross-linked duplex **6** derived from strand cleavage at the AP site. Lane 1: AP-containing duplex **4**. Lane 2: AP-containing duplex **4** treated with NaOH (200 mM at 60 ˚C for 30 min) to induce cleavage at the AP site. Lane 3: AP-containing duplex **4** incubated with spermine (Sp, 2 mM) in pH 7.4 HEPES buffer (50 mM, containing 100 mM NaCl) at 37 ˚C for 120 h, to generate a mixture of the authentic full-length cross-linked duplex **5** (Price, N. E.; Johnson, K. M.; Wang, J.; Fekry, M. I.; Wang, Y.; Gates, K. S. *J. Am. Chem. Soc.* **2014**, *136*, 3483-3490), the cross-linked duplex **6** derived from strand cleavage at the AP site (Yang, Z.; Price, N. E.; Johnson, K. M.; Wang, Y.; Gates, K. S. *Nucleic Acids Res.* **2017**, 45, 6486-6493), and short cleavage with 3'-phosphoryl, deoxyribose or PUA end groups. Lane 4: AP-containing duplex **4** was incubated in pH 7.4 HEPES buffer (50 mM, containing 100 mM NaCl) at 37 ˚C for 120 h, to generate a mixture containing the full-sized cross-linked duplex **5** (Price, N. E.; Johnson, K. M.; Wang, J.; Fekry, M. I.; Wang, Y.; Gates, K. S. *J. Am. Chem. Soc.* **2014**, *136*, 3483-3490), the strand-cleaved, cross-linked duplex **6** (Yang, Z.; Price, N. E.; Johnson, K. M.; Wang, Y.; Gates, K. S. *Nucleic Acids Res.* **2017**, 45, 6486-6493), the AP-containing duplex **4**, and products derived from spontaneous strand cleavage at the AP site in duplex **4**. Lane 5: AP-containing duplex **4** incubated in pH 3.5 citrate-phosphate buffer (17 mM and 33 mM, respectively, containing 100 mM NaCl) at 37 ˚C for 120 h. Lane 6: generation of slowly-migrating cross-linked DNA by incubation of duplex **1** in pH 3.5 citrate-phosphate buffer (17 mM and 33 mM, respectively, containing 100 mM NaCl) at 37 ˚C for 120 h. The band corresponding to cross-linked DNA co-migrates with authentic cross-linked duplex **5** and not authentic cross-linked duplex **6**. The DNA oligomers in the reactions were resolved by electrophoresis on a 0.4 mm thick 20% denaturing polyacrylamide gel at 500 V for 14 h and the Cy5-labeled fragments visualized by fluorescence imaging.

Figure S4

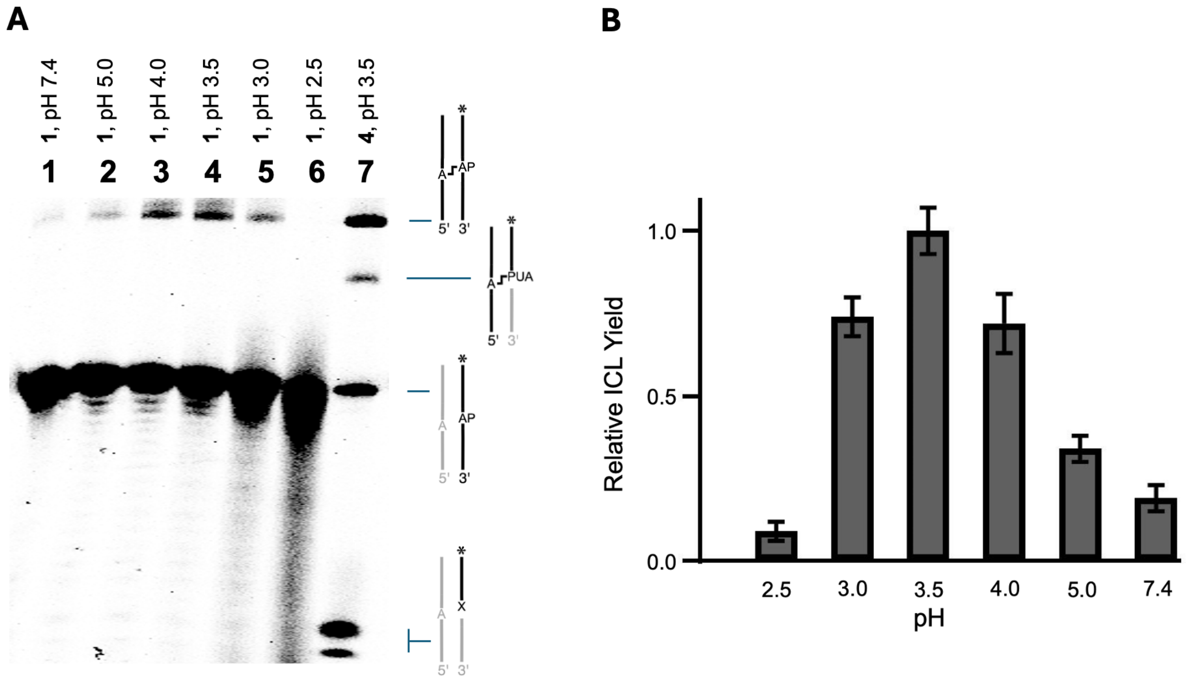

Figure S4. Gel electrophoretic analysis of ICL formation in DNA duplex **1** at different pH values. **Panel A**. Lane 1: AP-containing duplex **1** incubated pH 7.4 HEPES buffer (50 mM, containing 100 mM NaCl) at 37 ˚C for 120 h. Lane 2: AP-containing duplex **1** incubated in pH 5.0 citrate-phosphate buffer (17 mM and 33 mM, respectively, containing 100 mM NaCl) at 37 ˚C for 120 h. Lane 3: AP-containing duplex **1** incubated in pH 4.0 citrate-phosphate buffer (17 mM and 33 mM, respectively, containing 100 mM NaCl) at 37 ˚C for 120 h. Lane 4: AP-containing duplex **1** incubated in pH 3.5 citrate-phosphate buffer (17 mM and 33 mM, respectively, containing 100 mM NaCl) at 37 ˚C for 120 h. Lane 5: AP-containing duplex **1** incubated in pH 3.0 citrate-phosphate buffer (17 mM and 33 mM, respectively, containing 100 mM NaCl) at 37 ˚C for 120 h. Lane 6: AP-containing duplex **1** incubated in pH 2.5 citrate-phosphate buffer (17 mM and 33 mM, respectively, containing 100 mM NaCl) at 37 ˚C for 120 h. Lane 7: Incubation of the authentic, enzymatically-generated AP-containing duplex **4** in pH 3.5 citrate-phosphate buffer (17 mM and 33 mM, respectively, containing 100 mM NaCl) at 37 ˚C for 120 h, to generate a mixture containing the full-sized cross-linked duplex **5** (Price, N. E.; Johnson, K. M.; Wang, J.; Fekry, M. I.; Wang, Y.; Gates, K. S. *J. Am. Chem. Soc.* **2014**, *136*, 3483-3490), the strand-cleaved, cross-linked duplex **6** (Yang, Z.; Price, N. E.; Johnson, K. M.; Wang, Y.; Gates, K. S. *Nucleic Acids Res.* **2017**, 45, 6486-6493), the AP-containing duplex DNA **4**, and products resulting from spontaneous cleavage at the AP site in duplex **4**. The DNA oligomers in the reactions were resolved by electrophoresis on a 0.4 mm thick 20% denaturing polyacrylamide gel at 500 V for 14 h and the Cy5-labeled fragments visualized by fluorescence imaging. **Panel B**. Bar graph showing the relative ICL yields in duplex **1** at different pH values (same as Figure 4 in the main manuscript).

Figure S5

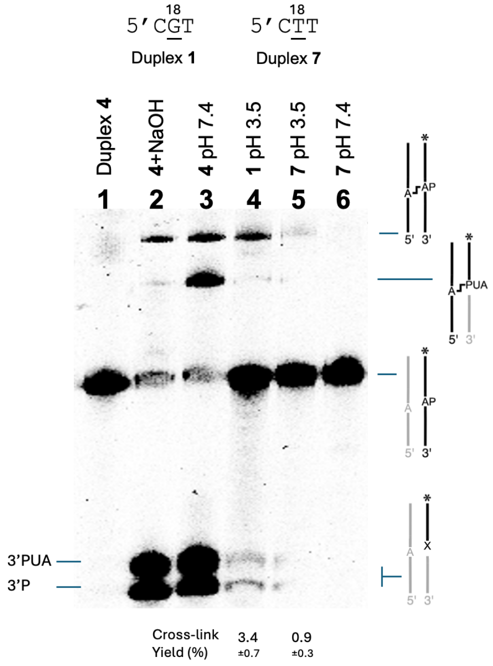

Figure S5. Depurination of G18 is a significant source of acid-induced ICL formation in duplex **1** at pH 3.5. The yield of acid-induced ICL generated in duplex **7** is low compared to that in duplex **1** (this is the full gel from which the data shown in Figure 5 of the main manuscript was obtained). Lane 1: AP-containing duplex **4**. Lane 2: AP-containing duplex **4** treated with NaOH (200 mM at 60 ˚C for 30 min) to generate products resulting from strand cleavage at the AP site. Lane 3: AP-containing duplex **4** incubated in pH 7.4 HEPES buffer (50 mM, containing 100 mM NaCl) at 37 ˚C for 120 h, to generate a mixture composed of the full-sized cross-linked duplex **5** containing the dA-AP ICL (Price, N. E.; Johnson, K. M.; Wang, J.; Fekry, M. I.; Wang, Y.; Gates, K. S. *J. Am. Chem. Soc*. **2014**, *136*, 3483-3490), the strand-cleaved, cross-linked duplex **6** containing the dA-PUA ICL (Yang, Z.; Price, N. E.; Johnson, K. M.; Wang, Y.; Gates, K. S. *Nucleic Acids Res.* **2017**, 45, 6486-6493), the AP-containing duplex **4**, and products arising from spontaneous strand cleavage of the AP site in duplex **4**. Lane 4: Duplex **1** incubated in pH 3.5 citrate-phosphate buffer (17 mM and 33 mM, respectively, containing 100 mM NaCl) at 37 ˚C for 120 h, to generate the acid-induced cross-linked duplex **5**. Lane 5: duplex **7** incubated in pH 3.5 citrate-phosphate buffer (17 mM and 33 mM, respectively, containing 100 mM NaCl) at 37 ˚C for 120 h. Lane 6: duplex **7** incubated in pH 7.4 HEPES buffer (50 mM, containing 100 mM NaCl) at 37 ˚C for 120 h. The DNA oligomers in the reactions were resolved by electrophoresis on a 0.4 mm thick 20% denaturing polyacrylamide gel at 500 V for 14 h and the Cy5-labeled fragments visualized by fluorescence imaging.
